# Structural homologies between phenformin, lipitor and gleevec aim the same metabolic oncotarget in leukemia and melanoma

**DOI:** 10.18632/oncotarget.16238

**Published:** 2017-03-15

**Authors:** Gábor Somlyai, T. Que Collins, Emmanuelle J. Meuillet, Patel Hitendra, Dominic P. D'Agostino, László G. Boros

**Affiliations:** ^1^ HYD, LLC for Cancer Research & Drug Development, Budapest, Hungary, European Union; ^2^ CignatureHealth Metabolic Clinic, Santa Monica, CA, USA; ^3^ EPIGENIX Foundation, El Segundo, CA, USA; ^4^ The University of Arizona Cancer Center, Tucson, AZ, USA; ^5^ Moores Cancer Center, University of California San Diego School of Medicine, La Jolla, CA, USA; ^6^ Department of Molecular Pharmacology and Physiology, Morsani College of Medicine, Hyperbaric Biomedical Research Laboratory, University of South Florida, Tampa, FL, USA; ^7^ Department of Pediatrics, University of California Los Angeles School of Medicine, Westwood, CA, USA; ^8^ Los Angeles Biomedical Research Institute (LABIOMED) at the Harbor-UCLA Medical Center, Torrance, CA, USA; ^9^ SiDMAP, LLC, Culver City, CA, USA; ^10^ UCLA Clinical and Translational Science Institute, Torrance, CA, USA

**Keywords:** imatinib, phenformin, deuterobolomics, lipitor, metformin

## Abstract

Phenformin's recently demonstrated efficacy in melanoma and Gleevec's demonstrated anti-proliferative action in chronic myeloid leukemia may lie within these drugs' significant pharmacokinetics, pharmacodynamics and structural homologies, which are reviewed herein. Gleevec's success in turning a fatal leukemia into a manageable chronic disease has been trumpeted in medical, economic, political and social circles because it is considered the first successful targeted therapy. Investments have been immense in omics analyses and while in some cases they greatly helped the management of patients, in others targeted therapies failed to achieve clinically stable recurrence-free disease course or to substantially extend survival. Nevertheless protein kinase controlling approaches have persisted despite early warnings that the targeted genomics narrative is overblown. Experimental and clinical observations with Phenformin suggest an alternative explanation for Gleevec's mode of action. Using ^13^C-guided precise flux measurements, a comparative multiple cell line study demonstrated the drug's downstream impact on submolecular fatty acid processing metabolic events that occurred independent of Gleevec's molecular target. Clinical observations that hyperlipidemia and diabetes are both reversed in mice and in patients taking Gleevec support the drugs' primary metabolic targets by biguanides and statins. This is evident by structural data demonstrating that Gleevec shows pyridine- and phenyl-guanidine homology with Phenformin and identical phenylcarbamoyl structural and ligand binding homology with Lipitor. The misunderstood mechanism of action of Gleevec is emblematic of the pervasive flawed reasoning that genomic analysis will lead to targeted, personalized diagnosis and therapy. The alternative perspective for Gleevec's mode of action may turn oncotargets towards metabolic channel reaction architectures in leukemia and melanoma, as well as in other cancers.

## INTRODUCTION

Gleevec's (Gleevec; Imatinib-mesylate, Glivec, STI-571) demonstrated anti-proliferative action in chronic myeloid leukemia ushered in the era of targeted therapies with claims that the drug blocks the constitutively active BCR-ABL tyrosine kinase thereby inhibiting phosphorylation of multiple downstream proteins of the mitogenic signaling pathways [1]. Gleevec's success in turning a fatal leukemia into a manageable chronic disease has been trumpeted in medical, economic, political and social circles because it is considered the first successful targeted therapy resulting from decade-long massive explorations of the genome [2]. Partly as a result of Gleevec's clinical success, genomic reductionism has almost completely overtaken medical thinking, dismantled model- and hypothesis driven research efforts, monopolized funding, and diverted medical education and therapy considerations towards personalized genomic platforms described as big sequencing data biology [3]. Omics approaches continue to dominate costly drug development efforts despite decades-long failures [4]. This collective enthusiasm for Gleevec and significant investments in omics with excessive economic, public, and political expectations, have persisted despite early warnings that the targeted genomic narrative is overblown [5, 6].

In their excellent paper Petrachi *et al*. [7] reiterate the significant therapeutic potential of Phenformin as a metabolic modulator of stem cell functions in therapy resistant melanoma, which aims the same metabolic oncotarget in leukemia and melanoma as an experimental evidence for an alternative explanation for Gleevec's mode of action. Previous *in vitro* studies have thrown into similar question whether the single-target kinase signal blocking mechanism is indeed the primary mechanism for STI-571's mode of action [6] using ^13^C-guided precise flux measurements, in a comparative multiple cell line study. Targeted ^13^C-glucose tracer fate association studies demonstrated the drugs' downstream impact on submolecular fatty acid processing and deuterium depleting metabolic events that occurred independent of Gleevec's molecular target. Clinical observations that hyperlipidemia and diabetes are both reversed in mice and in patients taking Phenformin and Gleevec support the drugs' primary metabolic targets [5, 8–10].

**Figure 1:**
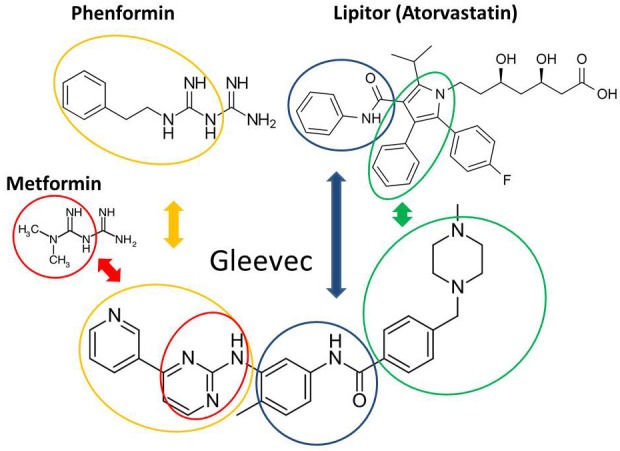
Structural homologies between Gleevec, Phenformin and Lipitor Pharmacodynamically modified (cleaved) Gleevec after binding (please see Figure 2 for binding homologies) has identical phenylcarbamoyl overlap with Lipitor (blue circles) that are produced by first round drug modification pharmacodynamics reactions involving oxidation and cleavage by cytochrome P450 family 3 subfamily A member 4 enzymes. Green cylinders indicate similar phenyl-pyrrole (Lipitor) and phenyl-piperazine (Gleevec) derivatives after the dihydroxyheptanoic acid residue is cleaved typically by β carbon oxidation. Orange circles show Phenformin and Gleevec sharing phenethylcarbamimidoyl- and pyridine-methyl-guanidine groups. Lactic acidosis is a common side effect of diguanidine drugs, which is also described in Gleevec [6] and Metformin [12, 31] treated cell cultures, as well as in Gleevec treated clinically drug resistant cells [22, 23].

The structural homology between Phenformin and Gleevec certainly links Petrachi *et al*.'s work in melanoma [7] to Gleevec's clinical efficacy in leukemia due to their shared metabolic effects rather than the generally accepted mechanism of BCR-ABL tyrosine kinase blockage. Gleevec's chemical structure (Chemical Abstract service (CAS) Registry Number: 152459-95-5) is that of an amino-phenyl guanidine derivative with significant structural, pharmacokinetics and pharmacodynamics homologies with Phenformin and atorvastatin (Figure 1). Like these two latter drugs Gleevec shows contextual inhibitory effect on thiolase, as well as various competitive pentose-phosphate as well as methyl-glutaryl- reductases involved in cholesterol and new cellular membrane synthesis, hence exhibiting an anti-proliferative action. Amino-phenyl class natural compounds [11], the guanidine derivative metformin [12] and Gleevec [6], which harbors both structural components, readily inhibit HMG-CoA reductase activity and thus divert acyl- and acetyl-residues towards complete fatty acid oxidation (β carbon) in mitochondria. Thereby contextual factors such as deuterium depleting ketogenic substrate oxidation, i.e. the complete breakdown of fatty acids in the expense of carbohydrates in mitochondria to yield metabolic water [13, 14] determine Gleevec's efficacy, regardless of a cell's BCR-ABL status. The complete ketogenic catabolic mechanism of Gleevec occurs in a drug-class specific manner, i.e. the same effects are observed with virtually all guanides and statins [15]. Striking pharmacodynamics similarities include that Gleevec undergoes first passage activation by gut/liver CYP3A4 (cytochrome P450 3A4; EC 1.14.13.97) to yield biologically active N-demethylated piperazine derivatives, Gleevec is highly (98%) bioavailable after a single oral dose, rapidly cleaved by oxidation to yield a guanide while structurally modified [16] so that only about 25% of the drug can be recovered from circulation unchanged after several passages. Gleevec also bears elimination in the bile and feces like biguanides and statins. Figure 1 shows pyridine- and phenyl-guanidine homology between Gleevec and Phenformin (Figure 1, orange circles) and identical phenylcarbamoyl structural homology of Gleevec with Lipitor (Figure 1, blue circles).

**Figure 2:**
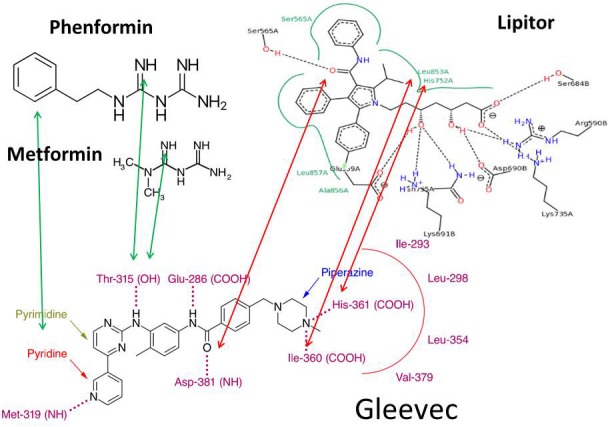
Gleevec's proton sharing binding properties *via* overlapping structural amino acid sites in BCR-ABL and Lipitor and Phenformin binding ligands Drug binding low affinity and high capacity hydrogen bonding architectures are shared among Lipitor, Phenformin, Metformin and Gleevec. Lipitor has similar proton bridging architectures by its structural homologies with Gleevec (red arrows). These include histidine, leucine, iso-leucine, as well as serine, as visualized by two dimensional protein−ligand complex architectures [19]. Gleevec's guanidine group binds to BCR-ABL through Threonine-315 (green arrows), which is similar to the binding of Metformin and Phenformin to the AMPK-γ subunit to induce conformational changes and promote the phosphorylation of Threonine-172 in AMPK. Stoichiometric modifications of Threonine-315 and Methionine-319 in BCR-ABL by the phenethylcarbamimidoyl- and pyridine-methyl-guanidine groups of Gleevec are evident. (Black dashed lines in Lipitor indicate hydrogen bonds, salt bridges, and metal interactions. The green solid line (not the arrows) shows hydrophobic interactions and green dashed lines show π-π and π-cation interactions, which are determined by geometric criteria as described in [19].

It is worth knowing that all analogues designed to treat Gleevec resistance share guanide structural similarities and target the same ATP pocket for binding, all of which result in remissions in chronic myeloid leukemia. Petrachi *et al*.'s work [7] may extend arguments that phenyl-guanidine homologies between Gleevec and Phenformin work through similar metabolic and ligand binding architectures targeting kinase domains *via* six hydrogen bond interactions [17]. Such hydrogen bonds involve the pyridine-N and backbone-NH of Met-318 and the hydroxyl aminopyrimidine side chain of Thr-315 [18]. Figure 2 summarizes Gleevec's proton sharing binding properties *via* distinct amino acid residues that are also known to bind Lipitor, Metformin and Phenformin in their own protein targets. These low affinity and high capacity drug binding architectures are evidently shared by Lipitor, Phenformin and Gleevec, which include histidine, leucine, iso-leucine, as well as serine, as specific amino acid ligands in two dimensional visual structures [19]. Gleevec's guanidine group binds to BCR-ABL through Threonine-315 (green arrow), which is similar to the binding mechanism of Metformin and Phenformin to the AMPK-γ subunit to induce conformational changes during the phosphorylation of Threonine-172 of AMPK. Stoichiometric modifications of Threonine-315 and Methionine-319 in BCR-ABL by the phenethylcarbamimidoyl- and pyridine-methyl-guanidine groups of Gleevec (Figure 2) induce metabolic rearrangements according to the deuterium depleting ketogenic substrate utilization metabolic phenotype, as described in ^13^C-glucose guided metabolomics studies [6, 12]. The tripartite mechanism using 1) Lipitor-like binding architectures to BCR-ABL with 2) a serine/threonine kinase targeting guanidine mechanism provides 3) anticancer and antidiabetic properties [20] to Metformin, Phenformin and Gleevec. Metabolic adaptation of BCR-ABL-negative Gleevec sensitive rat glioblastoma (C6) cells can be primed by hydroxycarbamide (hydroxyurea, HU) treatment for a more robust metabolic response [21], a structure also embedded in Gleevec by conditional oxidation of its guanide group (Figure 1, red circles). This underlines the significance of the primary metabolic mechanism of Gleevec's action in a broad range of cellular phenotypes by the alternate fueling of the TCA cycle according to a metabolic branching for acetyl-CoA in ketosis [21–24]. These studies demonstrate Gleevec's impact on fatty acid metabolic events that occurs independent of its molecular target. On the other hand Gleevec resistance is maintained in glycolytic cells with intact BCR-ABL by elevated glucose uptake and lactate production with small new fatty acid fractions and limited turnover. As a result, elevated glucose uptake with nonoxidative pentose processing and intense glycolysis, while lacking significant fatty acid turnover, are used as sensitive metabolic markers for early detection of Gleevec resistance in both BCR-ABL-positive and BCR-ABL-negative cells [22, 23]. More specifically, therapeutic concentrations of Gleevec (0.1-1.0 micromol/L) decreased glucose uptake while increasing the activity of the mitochondrial Krebs cycle with improved mitochondrial acetyl-CoA catalysis. Unlike standard chemotherapeutics, Gleevec, without cytocidal activity, reversed the Warburg effect by switching cells from glycolysis to mitochondrial acetyl-CoA breakdown while limiting glucose uptake and improving energy homeostasis [24].

*In summary*, the excellent paper by Petrachi *et al*. [7] that demonstrates the therapeutic potential of the biguanide class metabolic regulator Phenformin in melanoma lies close to pharmacokinetics, pharmacodynamics, structural and ligand binding homologies between Phenformin, Lipitor and Gleevec. These drugs therefore aim the same mitochondrial metabolic oncotarget as in leukemia. This work also points to the misunderstood mechanism of action of Gleevec, which is emblematic of the pervasive flawed reasoning that genomic analysis will lead to targeted, personalized diagnosis and therapy. It is evident that oncotargets are moving to the more reliable and phenotypically conserved metabolic arena. A conscious evaluation of the genomics era offered herein and sequencing efforts point again to inherently constructed failure paths in big biology [25] as well as severe conceptual shortcomings of personalized oncology and medicine [26–29]. The study of Petrachi *et al*. [7] opens directions from the narrow focus on genetic signaling mechanisms towards metabolically driven submolecular targets, in particular ketogenic metabolism's deuterium depletion in cytoplasmic and mitochondrial matrix water [13, 14], based on the clear structural and functional overlaps among targeted kinase inhibitors and metabolically positioned drugs for translational research and clinical medicine [30].
